# Effectiveness of Telecare Interventions on Depression Symptoms Among Older Adults: Systematic Review and Meta-Analysis

**DOI:** 10.2196/50787

**Published:** 2024-01-17

**Authors:** Man Wu, Chaoyang Li, Ting Hu, Xueyang Zhao, Guiyuan Qiao, Xiaolian Gao, Xinhong Zhu, Fen Yang

**Affiliations:** 1 School of Nursing Hubei University of Chinese Medicine Wuhan China; 2 Hubei Shizhen Laboratory Wuhan China

**Keywords:** telecare, depression, anxiety, quality of life, older adults, meta-analysis

## Abstract

**Background:**

Depression is the most common psychiatric disorder among older adults. Despite the effectiveness of pharmacological and psychological therapies, many patients with late-life depression (LLD) are unable to access timely treatment. Telecare has been shown to be effective in addressing patients' psychosocial issues, while its effectiveness in serving patients with LLD remains unclear.

**Objective:**

This study aimed to evaluate the effectiveness of telecare in reducing depression and anxiety symptoms and improving quality of life (QoL) in patients with LLD.

**Methods:**

Databases including the Cochrane Library, Web of Science, PubMed, Embase, and EBSCO were searched for randomized controlled trials (RCTs) evaluating the effectiveness of telecare for LLD from database establishment to December 28, 2022.

**Results:**

A total of 12 RCTs involving 1663 participants were identified in this study. The meta-analysis showed that (1) telecare significantly reduced depressive symptoms in patients with LLD compared to those in usual care (UC; standardized mean difference [SMD]=–0.46, 95% CI –0.53 to –0.38; *P*<.001), with the best improvement observed within 3 months of intervention (SMD=–0.72, 95% CI –1.16 to –0.28; *P*<.001); (2) other scales appeared more effective than the Patient Health Questionnaire-9 for LLD in telecare interventions (SMD=–0.65, 95% CI –0.96 to –0.35; *P*<.001); (3) telecare was more effective than telephone-based interventions for remote monitoring of LLD (SMD=–1.13, 95% CI –1.51 to –0.76; *P*<.001); (4) the reduction of depressive symptoms was more pronounced in patients with LLD with chronic conditions (SMD=–0.67, 95% CI –0.89 to –0.44; *P*<.001); (5) telecare was more effective for LLD in Europe and the Americas than in other regions (SMD=–0.73, 95% CI –0.99 to –0.47; *P*<.001); (6) telecare significantly reduced anxiety symptoms in patients with LLD (SMD=–0.53, 95% CI –0.73 to –0.33; *P*=.02); and (7) there was no significant improvement in the psychological components of QoL in patients with LLD compared to those receiving UC (SMD=0.30, 95% CI 0.18-0.43; *P*=.80).

**Conclusions:**

Telecare is a promising modality of care for treatment, which can alleviate depression and anxiety symptoms in patients with LLD. Continued in-depth research into the effectiveness of telecare in treating depression could better identify where older patients would benefit from this intervention.

## Introduction

Statistics show that the world’s population older than 60 years will double between 2015 and 2050, increasing from 12.0% to 22.0% [1]. With the rapid growth of the older population, late-life depression (LLD) has gradually emerged as a hot topic in the field of geriatric medical research. LLD refers to depressive disorders occurring in adults older than 60 years [2,3]. Research findings indicate a global prevalence of LLD of 28.4% [4], which could potentially be higher among individuals with concurrent physical ailments. As a geriatric syndrome with multifactorial etiology, LLD is highly associated with medical problems that pervade later life, including diabetes, hypertension, and dementia [2,5]. LLD is often chronic or recurrent and is associated with functional impairment, diminished health-related quality of life (QoL), and impaired social-psychological functioning [3,6]. A study confirmed that health care costs for patients with LLD were 43.0% to 52.0% higher for outpatient services and 47.0% to 51.0% higher when outpatient and inpatient services were combined, compared to those for individuals without LLD [7].

Despite its high prevalence and severe adverse outcomes, LLD is often overlooked and inadequately treated due to other complications resulting from aging-related issues. Psychopharmacotherapy and psychotherapy have been demonstrated to be effective for people with depression [6]; however, these treatments still have limitations, such as medical side effects and poor treatment adherence [8,9]. Due to mobility issues, geographic isolation, stigma associated with mental illness, and negative beliefs about treatment, older adults have limited access to health care or may be unwilling to seek help from health care institutions [10-12]. Additionally, underuse of professional mental health services, including low detection rates by health care providers and the lack of awareness among older patients regarding the severity of their condition [13,14], is also one of the factors that impede the treatment of LLD. Limited by these factors, only a minority of older adults receive appropriate treatment for depression. Therefore, there is an urgent need to study the clinical effectiveness of alternative therapies for depression, which are more socially acceptable and easily available.

In recent years, there has been increasing attention toward using telecare to support the management and well-being of mental health [15]. Telecare refers to the delivery of health care directly to users, typically in their own homes, supported by information and communication technologies such as telephone, videoconferencing, and applications [16,17]. Health care professionals can remotely provide consultation, assessment, and intervention services to patients [18]. These services include, but are not limited to, lifestyle monitoring, remote monitoring of vital signs for diagnosis, as well as long-distance assessment and education. The benefits of telecare are evident. Evidence suggests that as a promising strategy, telecare services can serve as a medium to overcome certain barriers, thereby enhancing mental health care and increasing opportunities to access evidence-based care under different conditions [19]. Particularly, telecare benefits older adults who are socially isolated or physically frail due to illness, disability, or other familial roles [17,20]. Currently, telecare has been widely used in the management of various chronic conditions among older adults, such as diabetes, hypertension, Parkinson disease, etc, yielding positive outcomes [21-23]. Depression is a commonly observed chronic condition among older adults, closely associated with an approximate 50% increase in chronic disease-related health care costs [24]. Given the significant impact of LLD on patients' QoL and its potential consequences on decreased productivity or suicide, ensuring continuity of care is imperative. Telecare has been proposed as an effective alternative to help bridge this treatment problem. Considering the complexity and severity of LLD, it is necessary to further explore whether telecare is effective in improving health outcomes for patients with LLD.

Previous reviews have assessed the evidence related to the use of telecare for managing mental health issues [11,25]. In the field of psychiatry, telecare has been found to significantly impact mental health outcomes in older adults, including reducing emergency visits and hospitalizations, as well as improving cognitive function [11]. However, the efficacy of telecare for depression is inconsistent. Some studies suggest the effectiveness of telecare in reducing symptoms of depression [11,26], while others indicate that the impact of telecare on improving depressive symptoms is limited, even yielding contradictory results [27,28]. Previous meta-analyses examining the effectiveness of telecare on depression have mostly focused on adult populations [25-27]. However, compared to other age groups, LLD is considered to be different [14]. Differences in study design, intervention methods, and treatment intensity may contribute to varying clinical outcomes in telecare treatments for LLD. Despite recent meta-analyses demonstrating significant efficacy of telemedicine in alleviating depressive symptoms among older adults, the evaluation of its evidence remains limited [29]. Due to inherent heterogeneity in inclusion criteria, interpretation of these results should be approached cautiously. The severe clinical outcomes and interfering factors often pose significant challenges in the treatment of LLD. Determining whether telecare management is effective for LLD is critical. It is unclear how effective telecare is in improving depression, anxiety symptoms, and QoL in patients with LLD. Therefore, this systematic review and meta-analysis explored the efficacy of telecare for LLD.

## Methods

This systematic review was conducted in accordance with the PRISMA (Preferred Reporting Items for Systematic Reviews and Meta-Analyses) guidelines (Multimedia Appendix 1) [30].

### Search Strategy

We conducted searches in Cochrane Library, Web of Science, PubMed, Embase, and EBSCO for randomized controlled trials (RCTs) published from the inception of the databases up to December 28, 2022, without any language restriction. MeSH (Medical Subject Headings) and free search terms were both used in the literature search. The search terms included “cell Phones,” “telemedicine,” “smartphone,” “mobile applications,” “mobile phone*,” “telephone*,” “telehealth,” “tele-healthcare,” “electronic health*,” “application*,” “m-health,” “messaging,” “depression,” “depressive disorder,” “depress*,” “Major depression,” “sadness,” “late-life depression,” “LLD,” **“**aged,” “elder*,” “geriatric,” “senior people,” “RCTs,” etc. All titles, keywords, and abstracts have been reviewed in accordance with our search criteria. In this study, these research articles were exclusively published in English, focusing on telecare interventions for LLD. The specific search strategy is shown in Multimedia Appendix 2.

### Study Selection and Data Exclusion

The inclusion criteria were the following: (1) studies were RCTs reported in full text with their title and abstract; (2) the average age of the study population was at least 60 years; (3) participants were diagnosed with depression in accordance with any established diagnostic criteria or with a score above a cutoff of any established depression rating scale at baseline; (4) the studies compare telecare (mobile phone, telephone, app, video, etc) participants with the control group receiving usual care (UC; routine, offline, or standard care); and (5) any health care professional providing care (ie, psychiatrists, family physicians, nurses, psychologists, etc).

Exclusion criteria were the following: (1) patients with manic or psychotic episodes or symptoms; (2) studies not related to the objective of this review and insufficient data, such as failure to report depression scale scores; and (3) books and studies without full text and studies in the format of abstracts of conference papers.

### Data Extraction

Two authors independently reviewed all the databases, with specific search strategies for the relevant articles (MW and CYL). The software EndNote X9 (Clarivate) was used to import all the references and remove duplicates. After removing duplicates, the relevance of the title and abstract of the articles was evaluated. Any disagreements were discussed until a consensus was reached. After screening the title and abstract, the articles were selected for the next step of a full-text review. The 2 authors screened the full-text articles independently (MW and CYL). Finally, eligible articles included in the study were processed based on inclusion and exclusion criteria. Any discrepancies that arose during the assessment were resolved by a third reviewer (FY). Two authors independently extracted data from the included studies and entered them into a predesigned data extraction form. Data extracted for this study included the following: first author, year of publication, country, sample size, mean age, intervention approach, duration, presence or absence of comorbid chronic conditions, depression degree, and outcome measurement tools (Table 1).

**Table 1:** Basic characteristics of the included studies (N=12; all are randomized controlled trials).

First author (year); country	Sample size, N (TC^a^/UC^b^)	Age (years), mean (SD)	Duration	Comorbid chronic diseases	Depression degree	Outcomes
Rollman (2009) [31]; United States	302 (150/152)	TC: 64 (10.8); UC: 64 (11.2)	Baseline, 8 months	Yes	Moderate	HAM-D^c^ and SF-36^d^
Aburizik (2013) [32]; United States	52 (29/23)	TC: 66.4 (7.9); UC: 64.1 (10.5)	Baseline, 10 weeks	Yes	Mild	PHQ-9^e^ and BDI^f^
Lee (2014) [23]; Korea	25 (12/13)	TC: 66.7 (7.9); UC: 65.4 (8.6)	Baseline, 6 months	Yes	Mild, moderate	CES-D^g^
Villani (2014) [33]; Italy	80 (40/40)	TC: 71 (4); UC: 73 (5)	Baseline, 12 months	Yes	Moderate, severe	PHQ-9 and STAI-6^h^
Pickett (2014) [34]; United States	124 (60/64)	TC: 69.1 (10.9); UC: 68.6 (10.7)	Baseline, 12 weeks	No	Mild	PHQ-9
O'Neil (2014) [35]; Australia	121 (61/60)	TC: 61.0 (10.2); UC: 58.9 (10.7)	Baseline, 6 months	Yes	Mild, moderate	PHQ-9, CDS^i^, and SF-12^j^
Gellis (2014) [36]; United States	94 (46/48)	TC: 80.1 (7.8); UC: 78.3 (6.9)	Baseline, 3 months, and 6 months	Yes	Mild, moderate	PHQ-9, HAM-D, and SF-12
Yang (2019) [37]; China	212 (107/105)	TC: 61.25 (8.60); UC: 60.85 (10.80)	Baseline, 12 months	Yes	Mild, moderate	HADS-D^k^ and SDS^l^
Naik (2019) [21]; United States	225 (136/89)	61.9 (8.3)	Baseline, 6 months, and 12 months	Yes	Moderate	PHQ-9
Dobkin (2020) [22]; United States	72 (37/35)	TC: 65.62 (9.76); UC: 64.80 (9.62)	Baseline, 3 months, and 6 months	Yes	Moderate	HAM-D, BDI, HAM-A^m^, and SF-36
Almeida (2021) [38]; Australia	200 (79/121)	≥65	Baseline, and 52 weeks	No	Mild, moderate	PHQ-9, GAD-7^n^, and SF-12
Koehler (2021) [39]; Germany	156 (79/77)	TC: 68.30 (9.13); UC: 64.34 (11.35)	Baseline, 12 months	Yes	Moderate	PHQ-9 and SF-36

^a^TC: telecare.

^b^UC: usual care.

^c^HAM-D: Hamilton Depression Rating Scale.

^d^SF-36: 36-Item Short Form Survey.

^e^PHQ-9: Patient Health Questionnaire-9.

^f^BDI: Beck Depression Inventory.

^g^CES-D: Center for Epidemiological Survey, Depression Scale.

^h^STAI-6: Spielberger’s State Trait Anxiety Inventory.

^i^CDS: Cardiac Depression Scale.

^j^SF-12: 12-Item Short Form Survey.

^k^HADS-D: Hospital Anxiety and Depression Scale.

^l^SDS: Zung Self-Rating Depression Scale.

^m^HAM-A: Hamilton Anxiety Rating Scale.

^n^GAD-7: 7-item Generalized Anxiety Disorder Scale.

### Quality Assessment

Two authors (MW and CYL) independently assessed the quality of the studies using the Cochrane Risk of Bias tool [40]. The assessment tool included 7 items (random sequence generation, allocation concealment, blinding of participants and personnel, blinding of outcome assessment, incomplete outcome data, selective reporting, and other bias), and authors judged each item individually as “low risk,” “high risk,” and “unclear risk.” The study was considered to be of high quality with a low risk score for at least 4 domains, of which 3 key areas had to be included (random sequence generation, allocation concealment, and incomplete outcome data). Consensus was reached by 2 authors (MW and CYL) through discussion with a third evaluator (FY).

### Statistical Analysis

Data were analyzed using Stata (version 16.0; StataCorp) and Review Manager (version 5.3; The Cochrane Collaboration). Intervention effects were estimated by calculating Cohen *d* standardized mean differences (SMDs) and 95% CIs [41]. All studies reported outcomes as continuous data. The Cochran Q statistic and *I*^2^ statistic were used to assess the statistical heterogeneity between selected studies. Random-effects models were used when study heterogeneity was high (*P*<.10; *I^2^*>50%); otherwise, a fixed-effects model would be used. When heterogeneity identified across studies was high, we further performed subgroup analyses to explore possible explanations for heterogeneity. Publication bias was measured using a funnel plot and Egger linear regression analysis, and *P*<.05 on the Egger test indicated statistically significant publication bias [42].

## Results

### Literature Search

**Figure 1:**
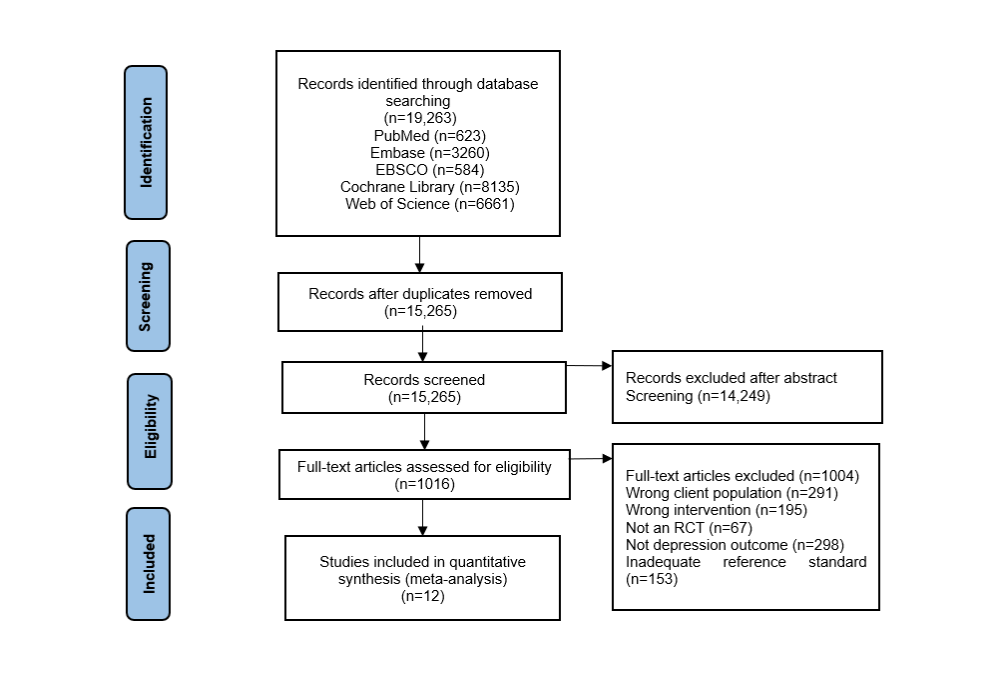
PRISMA (Preferred Reporting Items for Systematic Reviews and Meta-Analyses) flow diagram. RCT: randomized controlled trial.

The database search yielded 15,265 articles, of which 14,249 publications were excluded. A total of 1016 full-text articles were assessed for eligibility. Finally, only 12 studies were eligible for inclusion in this meta-analysis [21-23,31-39], all of which were RCTs published between 2009 and 2021. The PRISMA flow diagram is shown in Figure 1.

### Risks of Bias and Quality Assessment

**Figure 2:**
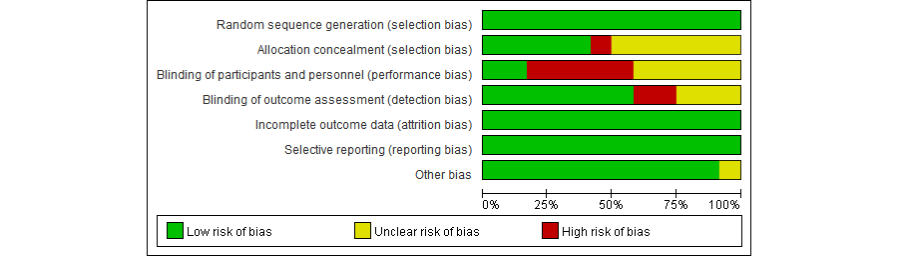
Overall risk of each type of bias.

**Figure 3:**
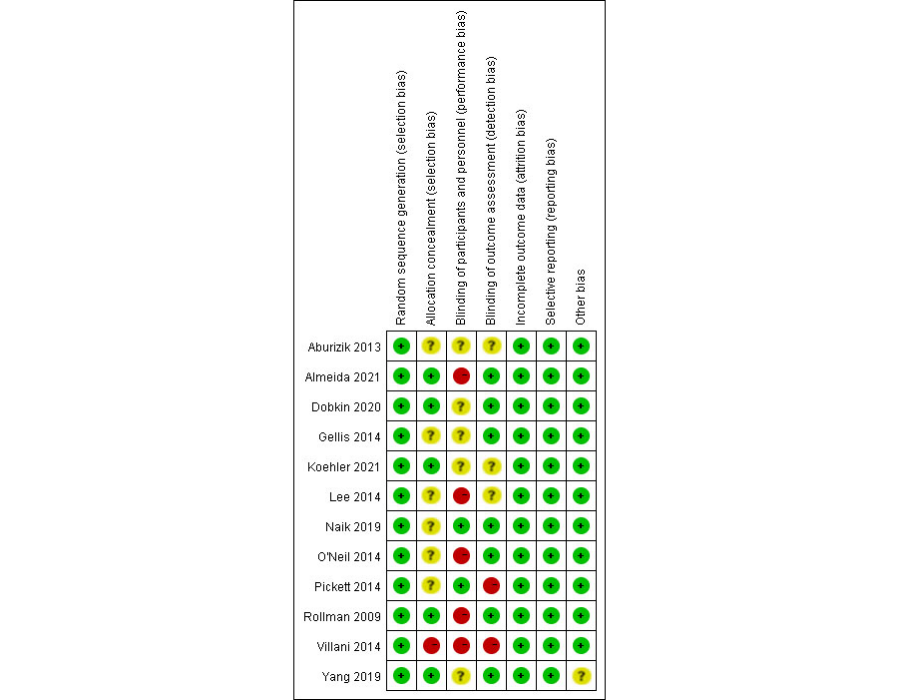
Risk of bias in each study.

Overall, the quality of the included studies was moderate, of which 5 (41.7%) were of high quality. These studies show that the main bias in the blinding of participants and personnel may be caused by the nature of the intervention measures. All 12 articles reported adequate random sequence generation and, therefore, had a low risk of bias in this regard. In addition, 5 studies reported allocation concealment, which is a low risk of bias. As for detection bias, the assessors were blinded in 7 studies, the presence of blinding was unclear in 3 studies, and 2 studies were not blinded. The risks of study attrition bias and reporting bias were both low. Other risks of bias were also low but were unclear in 1 study. The specific risk of bias and quality assessment results are shown in Figures 2 and 3 [21-23,31-39].

### Study and Patient Characteristics

The characteristics of the studies included are summarized in Table 1. A total of 1663 patients with LLD were involved, with an average age of over 60 years in each group. The sample size ranged from 25 [23] to 302 [31] participants. Studies were carried out across 6 countries, including the United States (n=6) [21,22,31,32,34,36], Korea (n=1) [23], Italy (n=1) [33], Australia (n=2) [35,38], China (n=1) [37], and Germany (n=1) [39]. Nine of these used telephone-based interventions, while the remaining studies used remote monitoring systems. Durations ranged from 10 weeks to 52 weeks. Depression, anxiety symptoms, and QoL were substantial influencing factors of treatment for older adults. Therefore, our primary outcome of interest was depression, and secondary outcomes were anxiety symptoms and QoL. Depression was evaluated using the Hamilton Depression Rating Scale, Patient Health Questionnaire-9 (PHQ-9), Beck Depression Inventory, Center for Epidemiological Survey, Depression Scale, Cardiac Depression Scale, Hospital Anxiety and Depression Scale, and Zung Self-Rating Depression Scale. Anxiety symptoms were assessed using Spielberger’s State Trait Anxiety Inventory, Hamilton Anxiety Rating Scale, and the 7-item Generalized Anxiety Disorder Scale. QoL was assessed using the 12-Item Short Form Survey and the 36-Item Short Form Survey. A higher score on the scales indicated better QoL and greater severity of depression and anxiety symptoms. The specific interventions are available in Multimedia Appendix 3.

### Depression Symptoms

A total of 12 RCTs involving 1663 participants were identified in this meta-analysis to calculate the effectiveness of telecare on depression, anxiety symptoms, and QoL in patients with LLD.

**Figure 4:**
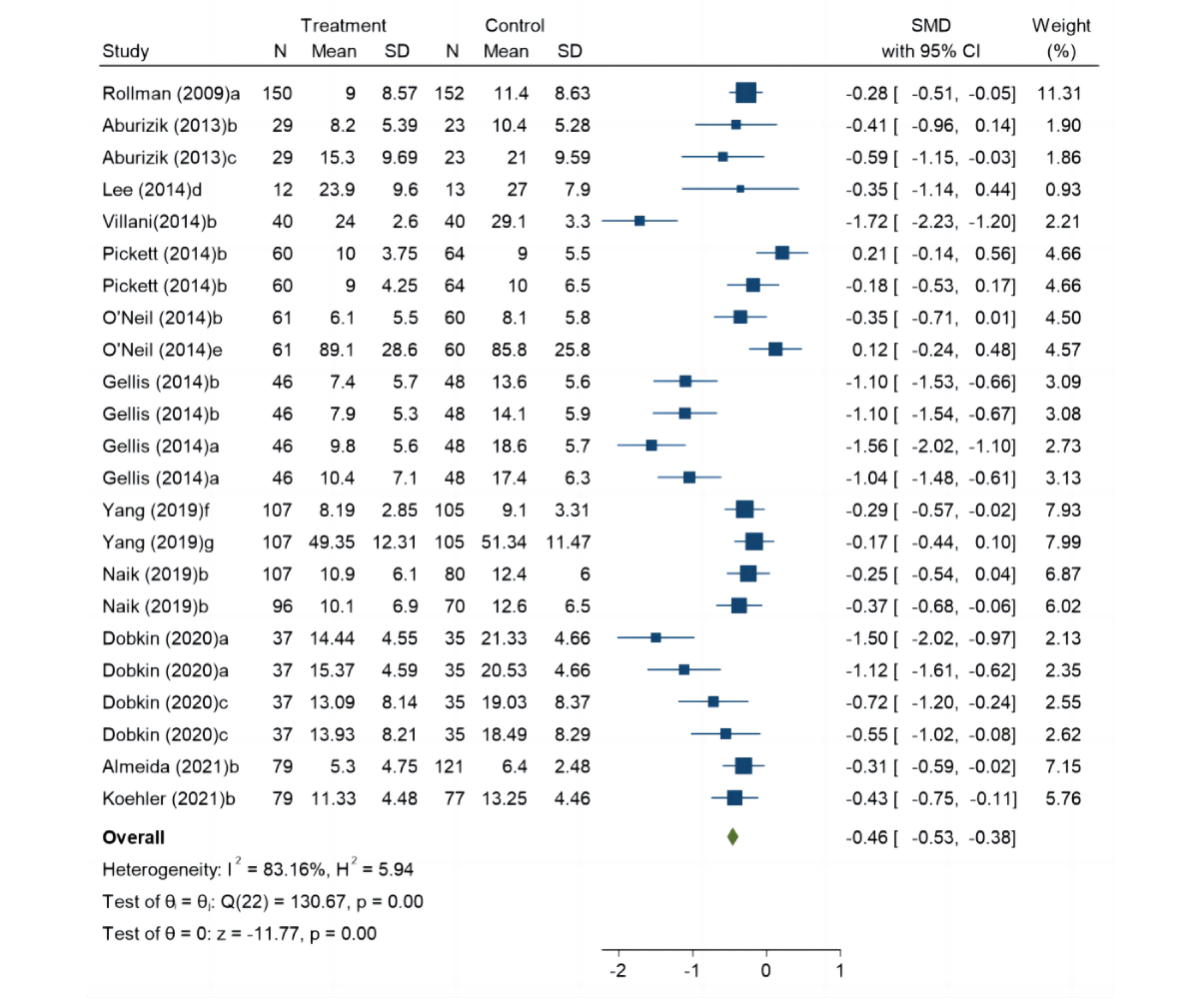
Forest plot for primary outcomes: depression. a: Hamilton Depression Rating Scale; b: Patient Health Questionnaire-9; c: Beck Depression Inventory; d: Center for Epidemiological Survey, Depression Scale; e: Cardiac Depression Scale; f: Hospital Anxiety and Depression Scale; g: Zung Self-Rating Depression Scale.

To compare the effects of telecare and UC in improving LLD, we included data from 12 of these studies. Our results show that telecare significantly reduced depressive symptoms in patients with LLD compared to those in UC (SMD=–0.46, 95% CI –0.53 to –0.38; *P*<.001). Fixed-effects model analysis revealed significant heterogeneity among the 12 included studies (*I*^2^=83.16%; *P*<.001; Figure 4) [21-23,31-39].

To address high heterogeneity, we performed subgroup analyses grouped by the type of scale (PHQ-9 or others), duration time (≤3 months or >3 months), device type (telephone-based or remote monitoring system), comorbid chronic diseases (presence or absence), and region (Europe and the Americas or others).

Random-effects models indicated that telecare significantly reduced depressive symptoms in patients with LLD compared to the UC participants (SMD=–0.59, 95% CI –0.80 to –0.38; *P*<.001). Results of subgroup analysis by duration showed that short-term (≤3 months) interventions (SMD=–0.72, 95% CI –1.16 to –0.28; *P*<.001) were more effective than long-term (>3 months) interventions (SMD=–0.52, 95% CI –0.75 to –0.29; *P*<.001); other scales (SMD=–0.65, 95% CI –0.96 to –0.35; *P*<.001) were more effective than the PHQ-9 (SMD=–0.53, 95% CI –0.83 to –0.22; *P*<.001); the remote monitoring system (SMD=–1.13, 95% CI –1.51 to –0.76; *P*<.001) was more effective than telephone-based interventions (SMD=–0.38, 95% CI –0.56 to –0.20; *P*<.001); the effect on patients with LLD with chronic diseases (SMD=–0.67, 95% CI –0.89 to –0.44; *P*<.001) was better than that on patients with LLD without comorbid chronic diseases (SMD=–0.10, 95% CI –0.41 to 0.20; *P*=.07); and telecare was more effective in Europe and the Americas (SMD=–0.73, 95% CI –0.99 to –0.47; *P*<.001) than in other regions (SMD=–0.22, 95% CI –0.35 to –0.09; *P*=.42; Table 2).

**Table 2:** Subgroup meta-analysis for patients with late-life depression.

Subgroups		Cohen *d* SMD^a^	95% CI	*P* value	Heterogeneity (*I*^2^; %)
Overall		–0.59	–0.80 to –0.38	<.001	86.42
**Duration**					
	≤3 months	–0.72	–1.16 to –0.28	<.001	86.91
	>3 months	–0.52	–0.75 to –0.29	<.001	84.72
**Type of scale**					
	PHQ-9^b^	–0.53	–0.83 to –0.22	<.001	87.13
	Others	–0.65	–0.96 to –0.35	<.001	86.21
**Device type**					
	Telephone-based	–0.38	–0.56 to –0.20	<.001	75.30
	Remote monitoring system	–1.13	–1.51 to –0.76	<.001	78.32
**Comorbid chronic diseases**					
	Presence	–0.67	–0.89 to –0.44	<.001	85.31
	Absence	–0.10	–0.41 to 0.20	.07	61.45
**Region**					
	Europe and the Americas	–0.73	–0.99 to –0.47	<.001	86.38
	Others	–0.22	–0.35 to –0.09	.42	0.00

^a^SMD: standardized mean difference.

^b^PHQ-9: Patient Health Questionnaire-9.

Meta-regression analysis showed that heterogeneity may not be related to the year of publication (*P*=.42), total sample size (*P*=.21), study area (*P*=.35), comorbid chronic disease (*P*=.47), duration (*P*=.75), and outcome measurement tools (*P*=.29). However, only the intervention device (*P*=.004) may have contributed to the heterogeneity.

### Sensitivity Analysis and Publication Bias

**Figure 5:**
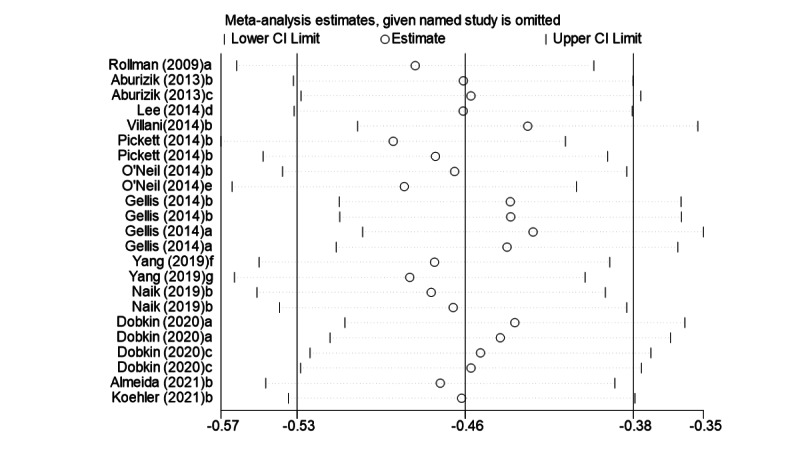
Sensitivity analysis of effect value (standardized mean difference).

**Figure 6:**
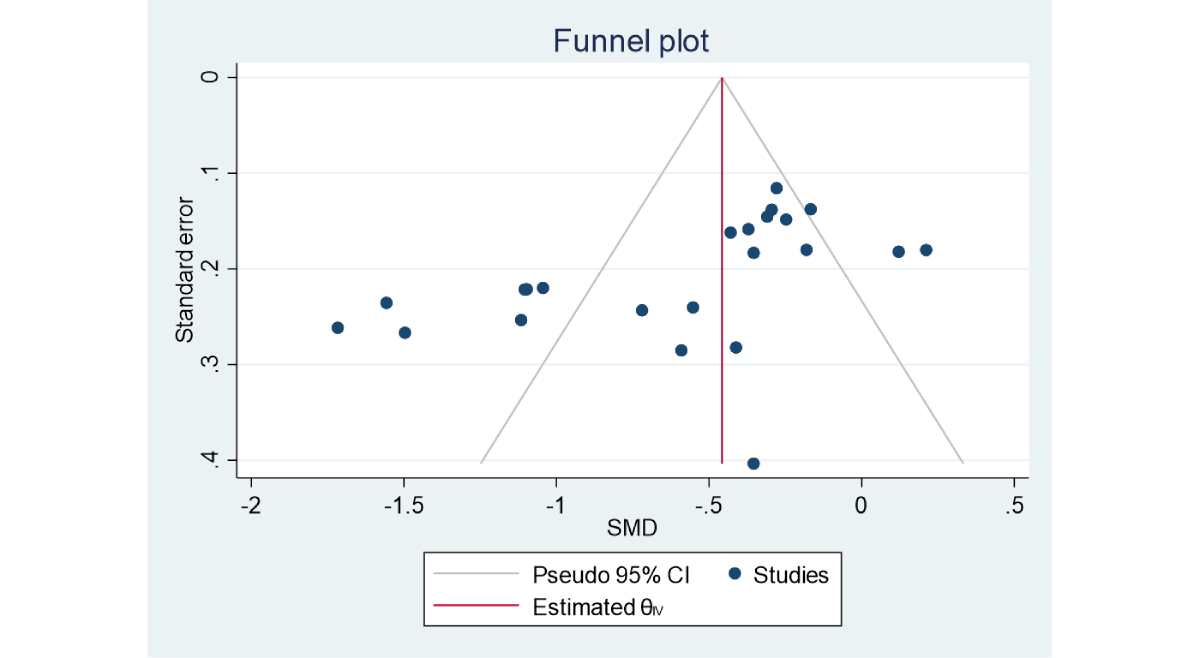
Funnel plot showing publication bias. SMD: standardized mean difference.

The stability and reliability of the results of this meta-analysis and potential factors contributing to heterogeneity were explored by sensitivity analysis to assess the effect of the data of each study on the combined effect value (ie, SMD). The results of the sensitivity analysis showed that excluding each study individually had no significant effect on the combined effect value, and the study results were stable and reliable (Figure 5) [21-23,31-39]. Publication bias was assessed using funnel plots and Egger test indicators. The funnel plot was symmetrically distributed on both sides (Figure 6), and the Egger test showed no significant publication bias (*P*=.05).

### Anxiety Symptoms

**Figure 7:**
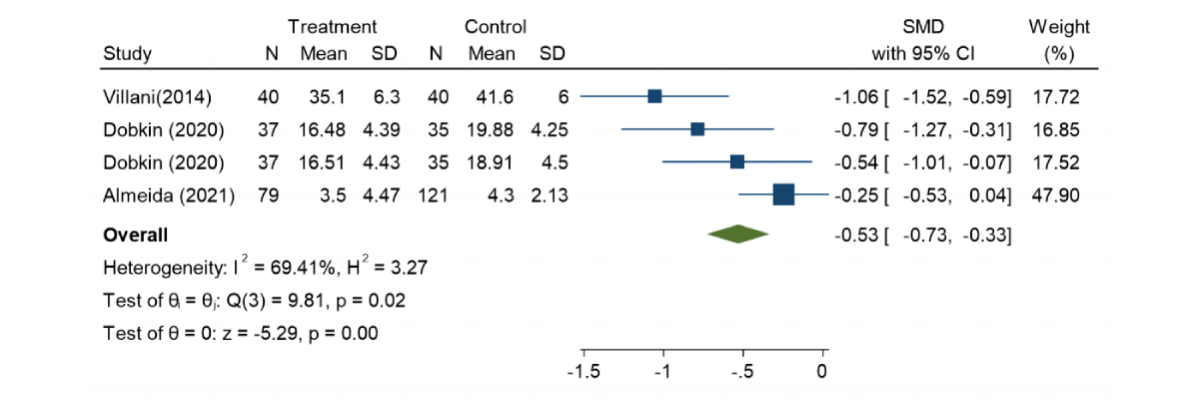
Forest plot of secondary outcome: anxiety.

To examine the efficacy of telecare in reducing anxiety compared with that of UC, we included 3 articles on patients with LLD. The results showed that telecare significantly reduced anxiety symptoms in patients with LLD (SMD=–0.53, 95% CI –0.73 to –0.33; *P*=.02; Figure 7) [22,33,38].

### QoL

**Figure 8:**
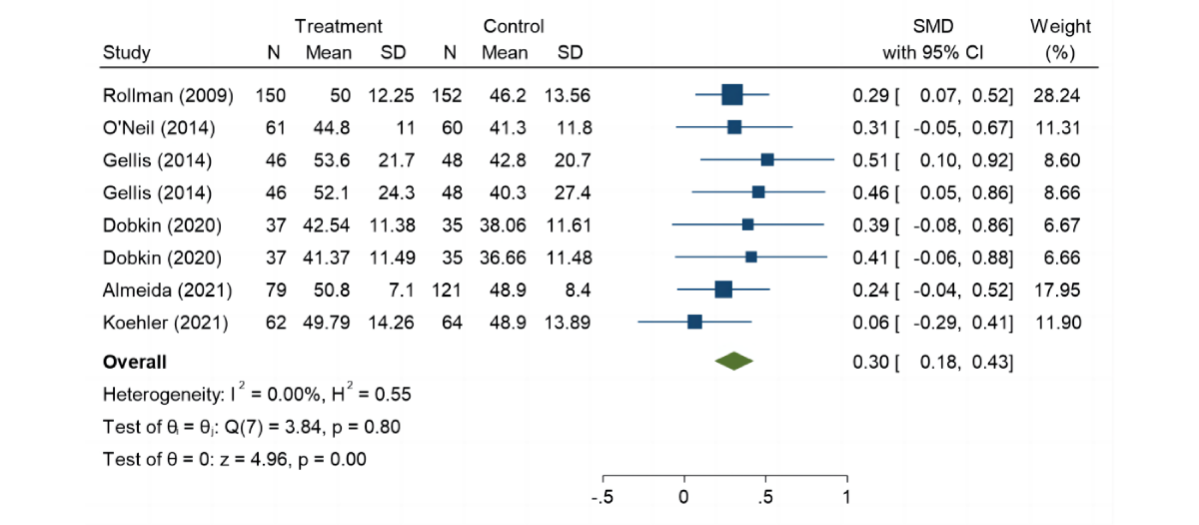
Forest plot of secondary outcome: quality of life.

Six studies assessed the mental components of QoL by using the Medical Outcomes Study Short Form survey. Our meta-analysis shows that the QoL of patients with LLD improved, but, overall, it was not significant (SMD=0.30, 95% CI 0.18-0.43; *P*=.80; Figure 8) [22,31,35,36,38,39].

## Discussion

### Principal Findings

This meta-analysis shows that compared with UC, telecare significantly reduces symptoms of depression and anxiety but has no significant effect on improving QoL in patients with LLD.

#### Primary Outcome Measures

The pooled results show that telecare has a significant effect on reducing depressive symptoms in patients with LLD, which is consistent with the findings of previous studies [25,26,29]. Apart from dealing with depression itself, the increased severity of LLD is also related to factors such as aging, chronic disease, and socioeconomic stress [5]. Telecare offers unique and innovative opportunities for treating depression symptoms in older adults. Patients with LLD can leverage the advantages of telecare to connect with health care professionals, overcoming geographical distance and physical limitations, thereby reducing the psychological burden of coping with the disease [43]. Furthermore, professional psychological support is crucial for patients with LLD, and it can encourage patients to express their feelings and release stress [44]. However, it is worth noting that despite telecare offering more possibilities for treating LLD, the complexity of the medical population makes it challenging. Telecare can provide greater coverage for health care, yet considerations such as individual needs of older patients or environmental backgrounds need to be factored in [45]. Currently, offering targeted telecare services to a large population of older adults in rural, remote, or underserved areas remains a challenge [10]. In particular, older adults face significant barriers in using telephone and internet connections during the COVID-19 pandemic [46]. As a result, telecare management may not be as effective for this population as for others. The size of the research effect will depend on the nature of the intervention and the quality of the study [47]. High-quality telemedicine will help older adults benefit both physically and mentally. Further investigation and more research are necessary.

Subgroup analysis indicates that the effectiveness of telecare in treating LLD can be influenced by measurement tools, durations, intervention devices, comorbid chronic conditions, and regions involved. In terms of depression measurement tools, other scales appear to be more effective than PHQ-9 (0.65% vs 0.53%), which may be related to measurement errors caused by differences in specific items and the generalizability of different measurement tools [48]. Results from durations of ≤3 and >3 months showed a reduction in depressive symptoms in patients with LLD, with short-term interventions proving to be more effective (0.72% vs 0.52%). Short-term interventions focus more on addressing specific issues or symptoms, producing immediate effects. For older adults, short-term interventions might be more readily accepted as long-term treatments could induce fatigue or a lack of patience. Our findings differ slightly from those of another study [49], which implemented more targeted interventions based on different treatment responses, confirming the more significant effectiveness of long-term interventions. Therefore, there is insufficient evidence to conclusively establish that telecare is necessarily superior in short-term intervention efficacy for LLD compared to long-term interventions. In fact, for depression management, a combination of short-term and long-term interventions is often required to deliver comprehensive and enduring support and management [50].

Subgroup analysis also found that remote monitoring systems appear to be more effective than telephone-based management (1.13% vs 0.38%). The remote monitoring system ensures timely and accurate transmission of patients' symptom information and data to health care professionals, enabling patients to receive effective treatments [51]. Telecare was more effective in patients with LLD with comorbid chronic conditions compared to those without such comorbidities (0.67% vs 0.10%). Co-occurrence of chronic medical conditions and depression is common. Evidence suggests that older adults with chronic illness are more likely to be affected by depressive symptoms than those without chronic illness [2,5,7]. Older adults with chronic conditions are more likely to seek medical care and adherent to treatment [52]. Therefore, while actively treating chronic conditions, there might be a degree of alleviation in depressive symptoms among older adults. Telecare was more effective in Europe and the Americas in improving depressive symptoms in patients with LLD compared than in other regions (0.73% vs 0.22%). The health care systems in Europe and the Americas are generally more developed, which may lead to more comprehensive support for telecare [53]. In low- and middle-income countries, the resources available for geriatric mental health care are considered severely inadequate [54]. Nevertheless, telecare is beginning to have an important impact on many aspects of health care in transitional countries [55]. 

#### Secondary Outcome Measures

Telecare has a positive effect on improving anxiety symptoms of patients with LLD. This result is consistent with findings from other studies [56]. Telecare offers a more convenient access method, allowing patients to receive treatment at home, thereby circumventing the inconvenience and anxiety associated with hospital visits [16,17]. Health care professionals can engage with patients more frequently through telecare, gaining insights into their symptoms and emotional fluctuations. This allows for adjustments in the treatment plan to effectively meet the unique needs of this population [18,57]. Additionally, the symptoms of anxiety and depression are often co-occurring [58], particularly among older adults. Due to the similarity between depression and anxiety symptoms, many treatment approaches are shared between the two. A recent meta-analysis suggests that psychotherapy delivered remotely is as effective as face-to-face therapy for anxiety disorder [59]. This evidence is based on outcomes obtained from different age groups. It may be more challenging to create a trusting relationship remotely than in person [60]. Older adults have negative views about health IT performing accurately and dependably, which will have a significant impact on the acceptance of telecare [61]. In brief, when using telecare for addressing emotional disorders in older adults, closer supervision and guidance might be necessary. Health care professionals need to distinguish the appropriateness of using telecare for communication and, in turn, individually tailor patient care.

We found that the mental component of QoL in patients with LLD improved after using telecare; however, this difference was not significant compared to that with the use of UC. This finding aligns with results from other studies [62,63]. Improving QoL is a comprehensive issue that includes not only improvements in health care but also social, psychological, and emotional factors [64]. Influenced by these factors, it is difficult to compare the results of QoL considering different contexts. Several results from RCTs with older adults using telemonitoring systems showed an improvement in the participants' QoL [65,66]; other telemonitoring RCTs could not achieve congruent results [67]. Improvements in QoL often require deeper interactions and personalized care. In particular, participants with mental disorders may benefit from individual and tailored solutions provided by general practitioners [68]. When using telecare, it is crucial to acknowledge that each subpopulation of marginalized older adults has differing strengths and needs. The studies we included focused more on managing the disease itself, which may weaken overall effectiveness. It is not easy to present telemedicine to the older population. The limitations inherent in older adults may lead to difficulties in receiving telecare, including the lack of technical literacy, equipment access barriers, cognitive function, etc [11]. These reasons could explain why telecare is not significant in improving the QoL of patients with LLD. The potential value of telecare in maintaining the QoL for individuals with LLD warrants further exploration. While this study did not reveal a positive impact of telecare on the QoL for patients with LLD, it has been established that telecare can assist patients with LLD in gaining more information about health services.

### Limitations

This study still had some limitations. First, most of the studies included in the review lacked sufficient measure detail, leading to irreversible bias. Our study mainly included 2 interventions based on telephone and remote monitoring to reduce this bias. Second, the measurement tools used in this study lacked standardization and heavily relied on self-reports from participants, introducing a degree of subjectivity and concealment that is not as rigorous as structured interviews. However, we attempted to validate the effectiveness of the results by using authoritative scales. Third, differences in the specific intervention methods, frequency, and content among the included studies may lead to clinical heterogeneity across different studies.

### Conclusions

Our meta-analysis shows that telecare has a positive impact on depressive and anxiety symptoms, despite high heterogeneity in depression symptoms. Therefore, studies with larger sample sizes and homogeneity were required to determine the effects of telecare in patients with LLD. Future research can continue to refine telecare systems and assess the specific needs of older vulnerable populations for more accurate evidence.

## Appendix

Multimedia Appendix 1 PRISMA checklist.

Multimedia Appendix 2 Search strategy.

Multimedia Appendix 3 The intervention content of the intervention group.

## Abbreviations

LLD: late-life depression
MeSH: Medical Subject Headings
PHQ-9: Patient Health Questionnaire-9
PRISMA: Preferred Reporting Items for Systematic Reviews and Meta-Analyses
QoL: quality of life
RCT: randomized controlled trial
SMD: standardized mean difference
UC: usual care
