# Supplementary material for: Effectiveness of Telecare Interventions on Depression Symptoms Among Older Adults: Systematic Review and Meta-Analysis
Source: JMIR Mhealth Uhealth. 2024 Jan 17;12:e50787. doi: 10.2196/50787 (PMC10831591; doi:10.2196/50787)
Supplement: Multimedia Appendix 2 [file mhealth_v12i1e50787_app2.doc]

**[Multimedia Appendix](https://www.ncbi.nlm.nih.gov/pmc/articles/PMC10422170/#app1) 2**

**Search strategy**

1. **Old people**

the aged OR

senior citizen* OR

senior people OR

old people OR

Geriatric OR

Older Adult OR

Elder* OR

the elderly OR

Aged OR

Aged people

**2.Depression**

Depression [MeSH] OR Depressive disorder [MeSH] OR Depressive disorder, major [MeSH] OR

Depress* OR

Depressed OR

Depression OR

Major depression OR

Major depressive disorder OR

MDD OR

Sadness OR

late-life depression OR

LLD

**3.phone**

Cell Phones [MeSH] OR Telemedicine [MeSH] OR Smartphone [MeSH] OR Mobile Applications [MeSH] OR Software [MeSH] OR

cellular phone* OR cell phone* OR mobile phone* OR telephone* OR mobile health OR telehealth OR telehealthcare OR electronic health* OR phone intervention OR app* OR mobile health OR Application* OR mhealth OR e-health OR iphone OR Messaging OR text reminder* OR text messages* OR phone based* OR WeChat

**4.Study design**

randomized controlled trial OR controlled clinical trial OR randomized OR randomly OR trial

**1# AND 2# AND 3# AND 4#**
