# Supplementary material for: Effectiveness of Telecare Interventions on Depression Symptoms Among Older Adults: Systematic Review and Meta-Analysis
Source: JMIR Mhealth Uhealth. 2024 Jan 17;12:e50787. doi: 10.2196/50787 (PMC10831591; doi:10.2196/50787)
Supplement: Multimedia Appendix 3 [file mhealth_v12i1e50787_app3.doc]

**[Multimedia Appendix](https://www.ncbi.nlm.nih.gov/pmc/articles/PMC10422170/#app1) 3. The intervention content of the intervention group**

| First author (year) | Intervention content |
| --- | --- |
| Rollman (2009) | Telephone program, provide basic psychoeducation about depression and its effect on cardiac disease, and describe treatment options. |
| Aburizik (2013) | Combined Psychotherapy and illness Management and 10 phone visits, included medication adherence, diet, health behaviors and symptom monitoring. |
| Lee (2014) | Problem-solving therapy,12 telephone-based PST ^b^ sessions to improve patients' problem-solving skills related to symptom management and lifestyle modification. |
| Villani(2014) | Telemonitoring system, remote monitoring of discharged patients' heart rate, weight, blood pressure, electrocardiogram, etc. through PDA, while setting monitoring frequency and time. |
| Pickett (2014) | Telephone-facilitated depression car protocol, induced for solving problems, behavioral activation, self-management, monitoring response to treatment, and countering premature discontinuation of medication. |
| O'Neil (2014) | Tele-health program, included motivational interviewing, goal setting, behavioral activation, and cognitive restructuring. |
| Gellis (2014) | Telehealth Education and Activation of Mood intervention included: telemonitoring, chronic illness and depression care management, and PST for comorbid depression. |
| Yang (2019) | Patients intensive telephone-based care program, included problem solving, education and instruction, emotional support, supervision, and promotion. |
| Naik (2019) | Telephone delivery of a collaborative goal-setting intervention, emphasis on nurse-patient communication and rapport establishment, using thoughts to improve wellness, diet, physical activity, medication management, and relaxation. |
| Dobkin (2020) | Telephone based cognitive behavioral therapy, induced coping skills to ameliorate depression, exercise, medication adherence, facilitating referrals and follow-through for ancillary therapies. |
| Almeida (2021) | Phone sessions, included collaborative care and self-managed BA ^a^ program, where psychologists guide patients over the phone to identify symptoms and manage behaviors. |
| Koehler (2021) | Telemedical intervention, usual care and a telemonitoring system to perform daily measurements blood pressure, their weight and so on. |

Note:BA: Behavioural Activation; ^b^ PST: Problem Solving Therap
